# H_2_O_2_/Ca^2+^/Zn^2+^ Complex Can Be Considered a “Collaborative Sensor” of the Mitochondrial Capacity?

**DOI:** 10.3390/antiox11020342

**Published:** 2022-02-09

**Authors:** Ester Sara Di Filippo, Franco Checcaglini, Giorgio Fanò-Illic, Stefania Fulle

**Affiliations:** 1 Department of Neuroscience Imaging and Clinical Sciences, University “G. d’Annunzio” of Chieti-Pescara, 66100 Chieti, Italy; es.difilippo@unich.it; 2 IIM—Interuniversity Institute of Myology, University “G. d’Annunzio” of Chieti-Pescara, 66100 Chieti, Italy; fanoillic@gmail.com; 3 Free University of Alcatraz, Santa Cristina di Gubbio, 06100 Perugia, Italy; franco.ceccaglini@libero.it

**Keywords:** mitochondrion, H_2_O_2_, Ca^2+^, Zn^2+^, oxidative eustress, oxidative distress

## Abstract

In order to maintain a state of well-being, the cell needs a functional control center that allows it to respond to changes in the internal and surrounding environments and, at the same time, carry out the necessary metabolic functions. In this review, we identify the mitochondrion as such an “agora”, in which three main messengers are able to collaborate and activate adaptive response mechanisms. Such response generators, which we have identified as H_2_O_2_, Ca^2+^, and Zn^2+^, are capable of “reading” the environment and talking to each other in cooperation with the mitochondrion. In this manner, these messengers exchange information and generate a holistic response of the whole cell, dependent on its functional state. In this review, to corroborate this claim, we analyzed the role these actors, which in the review we call “sensors”, play in the regulation of skeletal muscle contractile capacities chosen as a model of crosstalk between Ca^2+^, Zn^2+^, and H_2_O_2_.

## 1. Introduction

In this review, we want to explore in detail three messengers capable of collaborating and activating adaptive and response mechanisms in cooperation with the mitochondrion. In order to begin our journey inside the cell’s adaptive capacity, we tackle the concept of homeostasis understood as a cause that produces an effect which, in turn, cancels the cause. Furthermore, in this review, the general adaptation syndrome in order to describe the validity of the allostasis hypothesis, i.e., a hypothesis that sees the presence in the cell of a system of sensors capable of reading the functional situation of the cell and, at the same time, of evaluating the changes in the environment in which it lives, will be addressed. This would allow a modality of “anticipation” of cellular needs and, therefore, of a response which, if it is physiological, induces a situation of eustress. Conversely, if the cellular response does not have the strength to compensate for the alteration, this induces the formation of a state of distress.

Certainly, the generators of these responses cannot be single elements but multiple messengers capable of reading the environment, talking to each other, and having an “agora”, the mitochondrion. As it is known, based on the functions of the mitochondrion, we have reported that this intracellular organelle could be considered as a functional center, in which the messengers exchange information and generate a holistic response of the whole cell, dependent on its functional state.

Furthermore, the three most probable identified messengers, on the basis of their intracellular concentrations, compartmentalization, and ability shown to interact with one another in different pathophysiological situations, are H_2_O_2_, Ca^2+^, Zn^2+^. Furthermore, to corroborate this claim, we analyzed ion/ROS crosstalk in skeletal muscle focusing on the regulation of RYR and then its ability to generate force in the striated fiber.

Therefore, here, we will discuss in detail the role of the mitochondrion where the availability and flows of the three messengers are generated and where their interaction mechanisms can take place.

## 2. Oxidative Eustress and Distress

In living cells, several types of reactive species, such as reactive oxygen species (ROS), reactive nitrogen species (RNS), or reactive sulfur species (RSS), have a dual function. At low levels, they increase cellular capacity (oxidative eustress), while at high levels, they lead to a decline in cell viability until it dies (oxidative distress). The ROS/RNS/RSS levels inside the cells are strictly controlled by the balance between the rate of synthesis by ROS/RNS/RSS generating systems and the rate of removal through the non-enzymatic and enzymatic antioxidant systems. Excessive ROS/RNS/RSS production or the impairment of antioxidant status may disturb the cellular redox balance, inducing increases in oxidative stress (distress) in the intracellular comparts. However, both qualitatively and quantitatively, ROS play the most important role in this heterogeneous team [1].

ROS comprise a group of molecules derived from molecular oxygen, which is formed by oxidation–reduction (redox) reactions or electronic excitation. The chemical reactivity of the various ROS molecules is very different from each other and can easily reach orders of magnitude greater than 10. Therefore, the term “ROS” does not indicate a molecule, but rather a group of reactive substances even if it is possible to distinguish between species that derive from molecular oxygen, such as hydrogen peroxide (H_2_O_2_), and those that are the result of electronic excitation, such as hydroxyl radical (^−^OH) [2].

In conclusion, based on the enormous amount of data available today (in 2021, the Web of Science had more than 210,000 entries for ROS), it seems evident that the physiological levels of ROS support crucial cellular processes by also acting as messengers that can regulate intrinsic signaling pathways through direct and/or crosstalk mechanisms. In mammals, many comparts, such as the nervous system, or skeletal and cardiac muscles, are particularly susceptible to changes in redox status, developing a defensive or adaptive response depending on the concentration, source, and duration of pro-oxidative stimuli [3].

**Figure 1:**
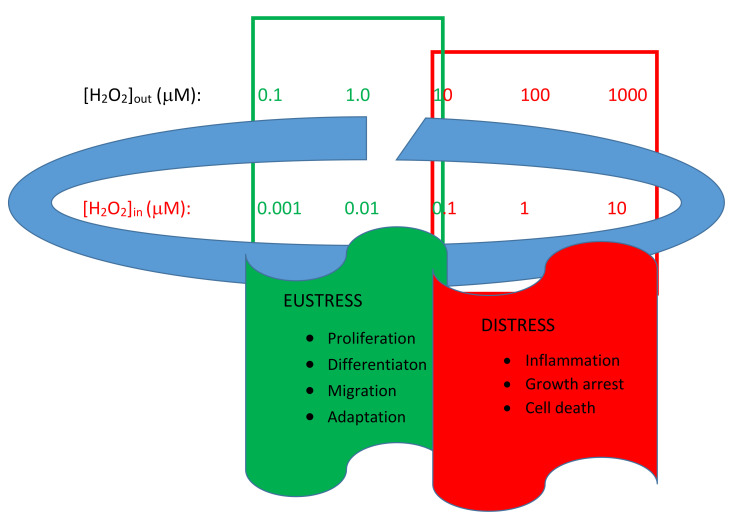
Homeodynamic space. The interpretative model of the regulation of the cellular redox state is based on the existence of a physiological range called “homeodynamic space”. The relative concentration of H_2_O_2_ could determine situations of eustress and distress. In the situation referred to as eustress, the intracellular H_2_O_2_ concentration is estimated to be 1–10 nM, while the cytosolic concentration is even lower (around 100 pM). The mitochondrial matrix has a peroxide concentration slightly higher than the cytosolic one (up to 20 nM), thus ensuring a gradient between the organelle and the cytoplasm. In the extracellular space, the H_2_O_2_ concentration is between 1 and 5 μM, thus ensuring a gradient of approximately 500 times between the outside and inside of the cell [10,11].

In a very recent review, Sies, a leading expert in the field of oxidative stress, proposed an interpretative model of the cellular redox state regulation based on the existence of a physiological range, called the “homeodynamic space”. In an open metabolic system, such as the cellular system, redox signaling requires continuous monitoring and fine-tuning of the steady-state redox set point. In the responses to oxidative stress, spatiotemporal control of redox signaling is achieved through the compartmentalized generation and removal of oxidants by short-term (non-transcriptional) and long-term (transcriptional/translational) homeostatic mechanisms [4]. The main actor in this modulation is H_2_O_2_. This is the key molecule in redox signaling, characterized by concentration differences of up to several orders of magnitude between the inside and outside of living cells and also between cellular compartments (Figure 1) [5].

Among the factors capable of altering H_2_O_2_ levels, but in different ways and different compartments, and thus changing the stable state from eustress to distress, all the factors that can act directly or indirectly on the peroxide synthesis and/or degradation systems should be included, while “long distance” messengers, such as hormones and cytokines, are less important [4].

One of the factors that can undoubtedly alter the situation of cellular eustress is hypoxia, which induces an increase in HIF, a transcription factor that acts as the main regulator of transcriptional responses to a decrease in oxygen levels [6]. Hypoxia, associated with increased O_2_^−^ (and subsequent H_2_O_2_ generation) due to inhibition of mitochondrial pathways, has been shown to activate redox signaling, which contributes to several systemic and cellular responses typical of the distressed state [7].

Another example of a factor that can regulate cellular oxidative stress is the FOXO protein. This transcription factor contributes to the maintenance of cellular and organismal homeostasis by integrating redox signals with other messengers. As observed with other redox signaling systems, this occurs through direct oxidation of cystein in FOXO members and oxidant-mediated tuning of upstream regulatory mechanisms [8].

In addition, p53, a transcription factor that governs responses to a variety of stresses associated with genomic instability, maintains cellular redox by regulating the expression of antioxidant genes. This role could support its tumor suppressor function in the tumor niche, which is typically pro-inflammatory and characterized by oxidative stress [9].

Again, redox signaling is coupled to nutrient stress. Both nutrient excess and deprivation are associated with the increased formation of oxidants, including H_2_O_2_, and modulation of the function of key nutrient sensors [1].

## 3. Homeostasis and Allostasis

A few years ago, P. Sterling published a paper that caused quite a sensation because it cast doubt on the role of the homeostasis mechanism (error correction by feedback) in maintaining the stability of the intracellular environment. Sterling proposed a new model: Allostasis. This mechanism assumes that efficient regulation must anticipate requirements and prepare to meet them before they arise. The advantages would be multiple: (i) errors are reduced; (ii) the response capabilities of different cellular components are matched; (iii) resources are shared between systems to minimize spare capacity [12]. However, this mechanism requires the presence of sensors that can not only detect the presence of certain agonists but also “read” the cellular metabolic situation in order to predict requests and thus avoid deficits.

Therefore, homeostatic systems need the presence of factors (cause) that alter the equilibrium inside the cell in order to generate a compensatory response (effect) which, in turn, should delete the cause of the perturbation (negative feedback). Obviously, homeostatic mechanisms require the presence of an alert system capable of detecting perturbing agents. Vice versa, according to the concept of Allostasis, the detection apparatus (sensors) is directed towards the cellular metabolic state and its requests through the analysis of the compartmentalization of the messengers already present in the internal medium. If this were true, the analysis of the internal environment obtained through changes in the concentrations and compartmentalization of the molecules acting as sensors should anticipate the requirements of the adaptive cellular response.

In our opinion, this approach does not invalidate the homeostatic principle, i.e., actions capable of maintaining the constancy of the internal milieu, but simply makes it more efficient. Indeed, all this takes place by adjusting the sensitivity of the sensors responsible for analyzing metabolic requirements so that any perturbation does not change the milieu equilibrium for a long time. We can assume the existence within the cell of systems both for receiving substances and/or episodic stimuli and analyzers of the cellular state. These systems are able to cooperate to make the living system’s response to perturbations induced by environmental variations more productive. ROS, and among them H_2_O_2_, could have the right characteristics to act as sensors of the intracellular situation. Indeed, they are the result of cellular metabolic activity, their concentration can vary by several orders of magnitude, and they are compartmentalized in the cells according to metabolic activities. So, it is evident that an accurate fine-tuning of this signaling mechanism is crucial for normal cell physiology, while a malfunction can lead to cell death.

## 4. Fine-Tuning of Mitochondrial Connection

**Figure 2:**
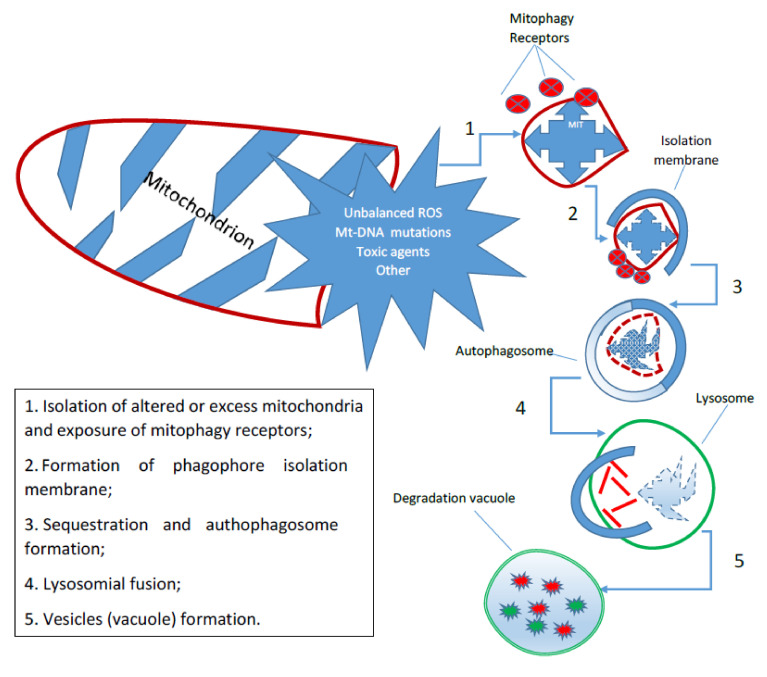
Scheme of Mitophagy. Mitophagy is a phylogenetically conserved mechanism that regulates the control of mitochondrial quality and quantity. It is triggered by specific receptors on the outer mitochondrial membrane or by ubiquitin molecules conjugated to proteins on the mitochondrial surface that lead to the formation first of autophagosomes and then degradation vacuoles.

The great adaptability of mitochondrial function to specific physiological or pathological conditions influences many cellular capacities, ranging from proliferation to apoptosis. Mitochondria are highly specialized organelles, but they are not only major producers of cellular energy. They contribute to various signaling pathways in continuous and dynamic interaction with their cellular environment. Therefore, a wide field of action requires a high level of structural and functional adaptation. Indeed, the mitochondria are constantly in motion and undergo fusion and fission processes, changing their shape and interaction with other organelles [13]. In particular, the clear question that emerges is which agents are able to tune the mitochondrial activity to the cell’s demand. We have just described one of these potential regulators, but it is not only the variation in H_2_O_2_ concentration and its compartmentalization that can explain the fine-tuning that most often associates mitochondrial activity with the metabolic needs of the cell. The mitochondrial activity is accurately regulated by the signaling between the inside (matrix) and outside (cytoplasm) of H^+^, K^+^, Na^+^ and Ca^2+^, Zn^2+^, etc. There are many possible interlocutors for a feasible “combined” action with H_2_O_2_ that can determine a fine regulation of mitochondrial activity and, thus, in the final analysis, of the entire cellular compartment. One of the most important mechanisms of the cell is autophagy, which is a catabolic process in which the constituents of cellular structures, including organelles, are degraded by lysosomes. Autophagy has multiple physiological functions, and alterations in its key steps have been linked to many important diseases, such as cancer and neurodegenerative disorders. It has also been shown that autophagy is one of the key factors regulating the lifespan of each mitochondrion and thus the total number of them available for cellular activities. The term *mitophagy* commonly refers to a selective form of autophagy, which ensures the preservation of healthy mitochondria by removing damaged or redundant mitochondria [14]. Mitophagy is a fundamental, phylogenetically conserved mechanism that regulates the control of mitochondrial quality and quantity. It is triggered by specific receptors on the outer mitochondrial membrane or by ubiquitin molecules conjugated to proteins on the mitochondrial surface that lead to the formation of autophagosomes (Figure 2). Mitochondrial mitophagy plays an important role in many cellular processes, from proliferation to differentiation and apoptosis. Alterations in the mechanism are associated with various pathophysiological conditions, such as aging, neurodegeneration, and cancer [15].

As damaged mitochondria are the main sources of reactive oxygen species, mitophagy is crucial for the control of mitochondrial quality and cellular health. Furthermore, controlling the number of mitochondria through mitophagy is vital for the balance of energy supply and demand in cells and whole organisms. For this reason, this mechanism is considered the core of the mitochondrial capacity to determine the organelle’s own functions, including ATP synthesis and H_2_O_2_ generation [16].

There are many factors, some of which are highly controversial, that are capable of interfering with the mitophagy mechanism [17]. Some of these play a secondary role, but others are considered to be crucial in controlling mitochondrial activity. Among the latter are surely some bivalent ions, such as Calcium, Zinc, and ROS, such as H_2_O_2_.

## 5. Work Hypothesis

Since the regulation of mitochondrial activity is fundamental in responding to changes in cellular needs, we think that the mitochondrion may be the seat of an alert system capable of responding accurately and, perhaps, anticipating the cell’s requirements.

In conclusion, we hypothesize that this cellular warning system may be the crosstalk between the most involved of the reactive oxygen species produced by the mitochondrion (H_2_O_2_) and some of the ions (Ca^2+^, Zn^2+^), which in turn are capable of using the mitochondrion as a functional exchange station.

The aim of this review is to analyze, using data from the literature, the validity of this hypothesis, and within this scenario, we will analyze the role that the indicated “sensors” play in the regulation of skeletal muscle contractile capacities chosen as a model of crosstalk between Ca^2+^, Zn^2+^, and H_2_O_2_.

## 6. H_2_O_2_ Properties

In all cells, the site that produces the most ROS as a result of electron loss during oxidative phosphorylation and molecular O_2_ reduction is the mitochondrion. This organelle, which has the fundamental role of producing energy for cellular requests, has also emerged as a participant in the regulation of cellular signaling, both under physiological and pathological conditions.

ROS, however, are also produced in many other cellular compartments, including phagosomes, peroxisomes, endoplasmic reticulum, cell membranes, etc. [18]. The mitochondrial production comprises a number of reactive species, including superoxide anion (O_2_^−^), the hydroxyl radical (^−^OH), and, notably, H_2_O_2_ [19]. The sources of H_2_O_2_ are linked to the activities of superoxide dismutase enzymes (SOD1, SOD2, SOD3), which are respectively located in the cytosol, in the mitochondrial matrix, and in extracellular space anchored to the extracellular matrix and cell surface. Other cellular compartments, such as the endoplasmic reticulum, also contribute, albeit to a lesser extent, to H_2_O_2_ production [20]. In this context, it should be borne in mind that numerous reduction reactions have been identified as sources of H_2_O_2_, although the main enzymatic generators are NADPH oxidases [21] and the mitochondrial respiratory chain, even if other oxidases may participate in peroxide synthesis [22].

The concentration of ROS in intracellular compartments is tightly controlled by scavenging mechanisms. O_2_ scavenging is established through dismutation to H_2_O_2_ by superoxide dismutases with Mn^2+^ (SOD2 in the mitochondrial matrix) or Zn^2+^/Cu^2+^ (SOD1 in the cytosol and SOD3 in the membranes). H_2_O_2_ scavenging occurs through reduction to H_2_O_2_ using both glutathione-dependent (dominantly) and non-glutathione-dependent reactions. Both pathways utilize NADPH (supplied by the pentose phosphate cycle in the cytosol or by transhydrogenation from NADH in the mitochondria) [23].

In addition, it must also be taken into account that the spatial distribution of H_2_O_2_ in cells and tissues is not uniform. There are substantial gradients, both between the extracellular and intercellular components and between the different intracellular compartments. This is also facilitated by the fact that there are specific aquaporins in biological membranes that allow the continuous translocation of H_2_O_2_ [24].

An exception to this general consideration is the mechanisms that regulate the Ca^2+^ and H_2_O_2_ relationships that occur at the endoplasmic reticulum/mitochondria interface, an area central to calcium signaling, organelle dynamics, and lipid biosynthesis. The mitochondrial and reticulum membranes also harbor sources and targets of ROS that can be measured using synthetic linkers. These linkers have demonstrated that this interface is home to a nanodomain of H_2_O_2_, which originates from the cristae but is generated by cytoplasmic Ca^2+^ peaks and exerts, in turn, positive feedback on ion oscillations [25].

In synthesis, current evidence shows that mitochondria play a prominent role in the regulation of cellular ROS homeostasis and that this mechanism is crucial for the maintenance of normal metabolic function. Under physiological conditions, mitochondrial ROS are involved in many signaling networks, starting with cysteine oxidation, which, mediated by H_2_O_2_, regulates cell survival signals. Hydrogen peroxide produced by the mitochondrion also acts on cell proliferation through various mechanisms, such as inhibition of tyrosine phosphatases [26], activation of cyclin-dependent kinase 1, and mitogen-activated MAP kinase [27]. Furthermore, in human fibroblasts, H_2_O_2_ regulates the diffusion within the cell of many growth factors and the transcription factor AP-1 [28].

The mitochondrial respiratory chain is the means by which the mitochondrion, by carrying out oxidative phosphorylation, fulfills the cell’s ATP requirements. It is made up of a series of electron transporters (complexes), most of which are integral inner membrane proteins containing prosthetic groups that can accept and donate one or two electrons. The electron-transporting protein complexes originally described are Complex I, Complex II, Complex III, and Complex IV, to which must be added two mobile electron transporters: Coenzyme Q and Cytochrome c. By using specific inhibitors of the different complexes, it has been shown that blocking Complexes I and III is capable of inhibiting ROS production without altering phosphorylation [29]. These compounds allowed a quantitative estimation of the overall contribution of mitochondria to cellular ROS production. They were successfully used to demonstrate that mitochondria are the most significant sources of H_2_O_2_ in cells, generated by the premature reaction of electrons with molecular oxygen before they reach Complex IV of the respiratory chain. In effect, between conventional and unconventional sites, there are 32 known ROS generators in mammalian cells, 16 of which reside in the mitochondria [30]. Mitochondria are also the seat of many antioxidant defenses, which are required to buffer cellular ROS by regulating the redox state. These same redox buffering networks also play another important role: the regulation of proteins through the reversible oxidation of cysteine switch sites by glutathione. This mechanism called S-glutathioneylation plays a key role in the control of different mitochondrial functions [31].

In conclusion, mammalian cells contain a system called “redoxome” that determines changes in the functional state of proteins (sensors and messengers) as a result of fluctuations in their oxidoreductive buffering capacity. These changes occur due to the redox modification of the cysteine thiols of the “redoxome” proteins in response to the balance of H_2_O_2_ and NADPH availability [32]. Due in part to H_2_O_2_’s pleiotropic properties, these pathways communicate with signaling cascades to modulate a wide range of cellular functions, including cell division and growth, immune cell motility and function, energy sensing, bioenergetics, and more [33].

## 7. Calcium Signaling and Mitochondria

As is well established after decades of scientific research on the subject, calcium ion signaling is crucial for cell function and survival. The main property of the calcium ion is to behave as an important second messenger involved in many intra- and extracellular signaling pathways [34].

**Figure 3:**
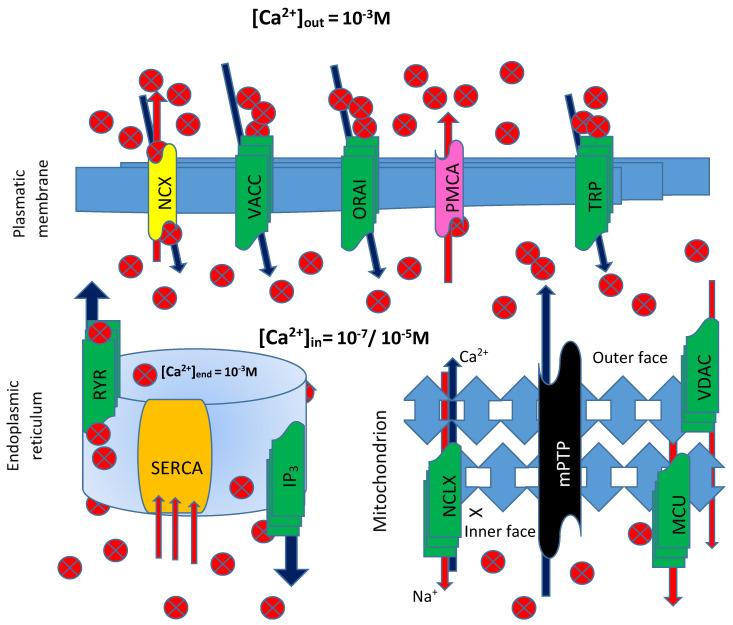
Calcium Signaling. Ca^2+^ ions enter into the cell through transient receptor potential (TRP) channels, voltage-activated Ca^2+^ channels (VACC), store-operated Ca^2+^ entry (ORAI) channels, or through modulator of Ca^2+^ homeostasis (NCX). The Na^+^/Ca^2+^ exchanger and the pump located on the plasma membrane (ATPase PMCA) extrude Ca^2+^ into the extracellular space from the cytosol. Inside the cell, another pump, the sarco/endoplasmic reticulum ATPase (SERCA), pumps Ca^2+^ into the lumen of the endoplasmic reticulum, from where it is released through IP_3_-receptor and Ryanodine-sensitive (RYR) channels. Mitochondrial Ca^2+^ uptake occurs through the voltage-dependent anion channel (VDAC) in the outer mitochondrial membrane and the mitochondrial Ca^2+^ uniporter complex, known as the MCU. Ca^2+^ is extruded under the control of the Na^+^/Ca^2+^ exchanger, encoded by the NCLX gene, and H^+^/Ca^2+^ antiporter. Ca^2+^ accumulation within the mitochondrial matrix provokes the formation of the mitochondrial permeability transition pore (mPTP), resulting in an uncontrolled release of Ca^2+^, apoptotic factors (AF), and reactive oxygen species (ROS).

The signaling modes used by the ion employ various procedures, such as buffers, pumps, and exchangers, on the plasma membrane and in internal stores. Ca^2+^ ions enter the cell through, among others, transient receptor potential (TRP) channels, voltage-activated Ca^2+^ channels (VACC), store-operated Ca^2+^ entry (SOCE) ORAI channels, or through modulator of Ca^2+^ homeostasis. The Na^+^/Ca^2+^ exchanger and the pump located on the plasma membrane (ATPase PMCA) extrude Ca^2+^ into the extracellular space from the cytosol. Inside the cell, another pump, the sarco/endoplasmic reticulum ATPase (SERCA), pumps Ca^2+^ into the lumen of the endoplasmic reticulum, from where it is released through IP_3_ receptor and Ryanodine-sensitive (RYR) channels [35] (Figure 3). In short, a sophisticated control network of channels, pumps, and cytosolic buffers controls numerous biological processes by properly regulating Ca^2+^ levels in various cell types. Electrical, hormonal, or mechanical stimuli trigger Ca^2+^ signals by facilitating Ca^2+^ entry across the plasma membrane or Ca^2+^ release from intracellular stores. To prevent the induction of damaging processes, Ca^2+^ actions are blocked by restoring resting Ca^2+^ concentrations within cellular compartments [36].

The mitochondria were the first intracellular organelles associated with Ca^2+^ management and sequestration. The high Ca^2+^ capacity of mitochondria is achieved primarily through the chelation of Ca^2+^ by phosphates in the mitochondrial matrix, forming amorphous Ca_3_(PO_4_)_2_ precipitates. Pioneering studies in the 1960s and 1970s demonstrated that mitochondrial Ca^2+^ uptake depended on the abrupt jump in mitochondrial membrane potential (Δψ~−180 mV) [37]. Mitochondria play a key role in regulating intracellular Ca^2+^ concentration by acting as a dynamic reservoir between the organelle matrix and the cytoplasmic environment. Therefore, the mechanisms that regulate Ca^2+^ entry into the mitochondria are fundamental in determining cell fate, as they are able to intervene in ATP synthesis processes by regulating the respiratory chain, as well as the mechanisms of mitophagy/autophagy and the mitochondrial pathway of apoptosis. Mitochondrial Ca^2+^ uptake occurs through the voltage-dependent anion channel (VDAC) in the outer mitochondrial membrane and the mitochondrial Ca^2+^ uniporter complex, known as the MCU channel, structurally constructed of four main subunits and a series of regulatory proteins. Ca^2+^ is extruded under the control of the Na^+^/Ca^2+^exchanger, encoded by the NCLX gene, and of a H^+^/Ca^2+^ antiporter, whose identity is still debated [38]. Overwhelming Ca^2+^ accumulation within the mitochondrial matrix provokes the formation of the mitochondrial permeability transition pore (mPTP), resulting in an uncontrolled release of Ca^2+^, apoptotic factors, and reactive oxygen species. The low affinity of the MCU complex, coupled to the activity of the efflux systems, protects cells from continuous futile exchanges of Ca^2+^ across the inner mitochondrial membrane and consequent massive energy dissipation. The dynamic interaction between these structures modulates MCU channel activity after detecting local changes in [Ca^2+^]_i_, ROS, and other environmental factors [39].

Therefore, the mitochondria can decode Ca^2+^ signals to meet the energy demands of the cell. In addition, the ability of mitochondria to transfer Ca^2+^ between different areas of the cell or to buffer cytosolic Ca^2+^ through the coordination of Ca^2+^ uptake and extrusion allows modeling the cytosolic Ca^2+^ signaling. Mitochondria can also buffer Ca^2+^ in the proximity of store-operated Ca^2+^ entry, SOCE, which are encoded by ORAI proteins and regulated by the Ca^2+^ sensor, stromal interaction molecule (STIM). In this case, the consequence is the attenuation of Ca^2+^ dependent inactivation of SOCE and the increase of Ca^2+^ entry into the cell [40]. Changes in mitochondrial Ca^2+^ uptake have been implicated in pathological conditions affecting multiple organs, including the heart, skeletal muscle, and brain. In cancer, cells modulate mitochondrial Ca^2+^ homeostasis ([Ca^2+^]_mit_) by altering the expression and function of mitochondrial Ca^2+^ channels and transporters required for mitochondrial Ca^2+^ uptake and extrusion. Regulated increases in [Ca^2+^]_mit_ are required for the activity of several mitochondrial enzymes, and this, in turn, regulates metabolic flux, ATP synthesis, and ROS generation. Alterations in both [Ca^2+^]_mit_ and ROS are characteristic of many cancers. A persistent high concentration of H2O2 is a known driver of pro-tumor redox signaling, resulting in the activation of pathways involved in cell proliferation and metabolic alterations, and disease-specific adaptations [41].

## 8. Zn^2+^ Signaling and Mitochondria

Zinc has many physiological functions in the cell, so its intracellular level is tightly controlled. Total Zn^2+^ concentration in human cells is in the range of several hundred micromolar (µM), with most Zn^2+^ being bound to proteins or sequestered into intracellular organelles [42]. Zn^2+^ is tightly regulated in a healthy cell where labile or free Zn^2+^ is maintained in the picomolar (pM) range because free Zn^2+^ is toxic to the cells. When zinc levels rise to nanomolar to micromolar levels, the cell undergoes zinc-induced cytotoxicity [43]. There are intracellular stores of Zn^2+^ represented, for example, by mitochondria, which store zinc in cells in a physiological state [44].

If the situation evolves towards a pathology as in ischaemic states, the regulation of zinc in the cell and mitochondria becomes unbalanced, eventually leading to high ROS generation [45].

Zinc homeostasis, including waves and sparks, observed using fluorescence imaging of cytosol Zn^2+^ variations, is closely associated with many processes, both physiological and pathological [46]. Indeed, these variations are involved in many regulatory mechanisms both intracellularly and between different cells. With the exception of some cellular compartments that maintain fairly stable Zn^2+^ levels, many physiological processes involving multiple organelles are also associated with Zn^2+^ fluctuations. In particular, an increasing number of evidences suggests that dynamic changes in intracellular Zn^2+^ are associated with processes such as autophagy and, via ROS, mitophagy [15].

Changes in mitochondrial morphology have great significance for cellular health; fusion is prevalent over organelle fission in healthy cells. In cells under precarious conditions and with a high level of fission mechanisms, mitochondria enter the mitophagic phase very early. It should be noted that an increase in the intracellular concentration of the Zn^2+^ can lead to mitochondrial stress by activating morphologies of mitochondrial damage and ROS generation [43].

Zn^2+^ is not only a co-factor for many enzymes involved in the physiological role of the antioxidant defense system but also protects cells against oxidative damage through stabilizing the homeostasis of several intracellular pathways. Among its activities, it plays an important role in restoring impaired energetic metabolism via the stabilization of membranes’ ionic homeostasis as well as mediating the phosphorylation and oxidation of several proteins, kinases, and enzymes [47].

Studies also have shown that Zn^2+^ plays an important role in the conversion of two superoxide radicals to hydrogen peroxide and molecular oxygen, reducing the toxicity of ROS [45]. This process occurs thanks to the triggering of a compensatory mechanism directly induced by the intracellular increase in ROS/RNS levels which, in turn, stimulate the release of Zn^2+^ from intracellular deposits, increasing the transient level of the ion with consequent activation of its antioxidant capacity [48]. Correspondingly, through the contribution of elevated ROS/RNS to the damage and dysfunction in cardiomyocytes, one can interpret why there is a close relationship between increased intracellular labile Zn^2+^ levels and deleterious changes in several signaling pathways in the heart [49].

Studies have demonstrated that any defect in the normal processes controlled by mitochondria can lead to abnormal ROS production and, therefore, high oxidative stress and lack of ATP. Experimental studies in mammalian cardiomyocytes and human heart tissue showed that Zn^2+^-transporters, under physiological conditions, localize to mitochondria besides sarco(endo)plasmic reticulum and Golgi. The protein levels, as well as the functions of those transporters, can re-distribute under pathological conditions. Therefore, they can interplay among organelles in cardiomyocytes to adjust a proper intracellular labile Zn^2+^ level [49].

In conclusion, both experimental and clinical studies show that alteration to Zn^2+^ homeostasis leads to deep changes in the functional state of the cell that can ultimately compromise its existence. However, there is much evidence pointing to a more plastic role for transient intracellular Zn^2+^ levels in mammalian cells capable of inducing opposite effects on cellular metabolism [50]. Indeed, there are strong indications that Zn^2+^ also plays a role as a protector of cellular health in opposition to oxidative damage [51]. From this point of view, it is not surprising that Zn^2+^ acts by inhibiting the enzyme nicotinamide adenine dinucleotide phosphate oxidase (NADPH-Oxidase), a pro-oxidant enzyme, and induces metallothionein synthesis [52]. However, most data in the literature point out that high intracellular Zn^2+^concentrations are toxic to cardiomyocytes as well as other cells.

Correspondingly, it is reported that an optimal ratio of cytoplasmic transient Zn^2+^ level and Ca^2+^ concentration in the cytosol and mitochondria has a significant mitochondrial-mediated antioxidant action. Under physiological conditions, the intracellular level of Zn^2+^ in ventricular cardiomyocytes is less than 1 nM in both rat and rabbit [53].

Under pathological or parapathological conditions, such as aging, as well as acute exposure to oxidants, its level can increase by two to 30-fold [54].

It should also be noted that, in muscle cells, an increase in intracellular Zn^2+^ concentration can, in turn, lead to Ca^2+^ dyshomeostasis, impaired excitation–contraction coupling, and mitochondrial dysfunction. These alterations will result in a major increase in ROS and/or RNS production, apoptosis, and/or cellular necrosis in the affected cells [55].

## 9. Crosstalk H_2_O_2_/Ca^2+^/Zn^2+^

Calcium and ROS crosstalk between endoplasmic reticulum and mitochondria. The endoplasmic reticulum (ER) is a major site of calcium storage. Calcium from ER cisternae flows mainly through calcium release channels, such as inositol 1,4,5-trisphosphate receptors (IP_3_R) and ryanodine receptors (RyR). These channels are accumulated in ER membrane regions, which associate with the mitochondrial outer membrane, by mediating the communication between the ER and mitochondria.

Calcium ions from the cytoplasm enter the mitochondria through voltage-dependent anion channels or calcium uniporters. High levels of calcium stimulate respiratory chain activity leading to higher amounts of ROS. In addition, ROS can target ER-based calcium channels leading to increased release of calcium ions and further increased ROS levels. Increased ROS and calcium load can open the mitochondrial permeability transition pore, resulting in the release of pro-apoptotic factors [56].

In particular, in organs, such as heart muscle, it has been shown that Zn^2+^ is released into the cytosol during the cardiac excitation–contraction cycle in a manner dependent on both Ca^2+^ concentration and redox state. This, in turn, can trigger H_2_O_2_ production through the induction of changes in the properties of metallothioneins [57].

In cardiomyocytes, it has also been shown that a significant Ca^2+^-dependent increase, over physiological level, in the intracellular concentration of Zn^2+^, is able to induce a marked increase in the cristae area of the mitochondrial matrix with the presence of clustering. This picture is often associated with an increase in the number of lysosomes, indicating a phase of active mitophagy in the cells. In conclusion, an increase in cytoplasmic free Zn^2+^ induced from Ca^2+^ alteration leads to an increase in ROS presence, and this fact is capable of altering mitochondrial function in cardiomyocytes [58].

Recent studies have highlighted that Zn^2+^ and ROS signaling systems are intimately integrated such that Zn^2+^-dependent regulation of components of ROS homeostasis might influence intracellular redox balance and vice versa. Zn^2+^-induced cell death is accompanied by increased levels of ROS and is attenuated by various antioxidant mechanisms. On the one hand, a number of ROS-generating and antioxidant systems of living cells have been shown to be Zn^2+^ dependent [59].

In an interesting paper published by Kira G. Slepchenko et al., it was shown that both Zn^2+^ and ROS accumulate during hypoxic-ischemic stress, using fluorescent probes in HeLa cells. In particular, two different increases in Zn^2+^ were observed during a time-course of thirty minutes stimulated by oxygen and glucose deprivation. The first increase was transient, followed by a latent phase during which Zn^2+^ levels fell, returning almost to baseline in 40% of cells while remaining sustained in 60% of cells. A second Zn^2+^ increase (sustained plateau), called Zn^2+^ overload, showed up in the second part of the experiment. Increases were not observed when Zn^2+^ was removed by treatment with TPEN (a Zn^2+^ chelator) or thapsigargin, an agent that depletes Zn^2+^ from intracellular stores, indicating that Zn^2+^ was coming from intracellular stores.

If mitochondria were damaged with FCCP (a mitochondrial uncoupler), the second Zn^2+^ increase decreased dramatically, indicating that the plateau was a consequence of mitochondrial ion release. The two Zn^2+^ increases preceded two ROS increases. Removal of Zn^2+^ reduced or delayed ROS generation, indicating that Zn^2+^ contributes to mitochondrial ROS generation [45].

Interestingly, transient levels of Ca^2+^ and Zn^2+^ have also been implicated in neurodegenerative processes during acute ischemia. In CA1 pyramidal neurons subjected to oxygen and glucose deprivation, Zn^2+^ increases precede and contribute to the onset of intracellular Ca^2+^ increases (Ca^2+^ deregulation), which are causally linked to the loss of mitochondrial membrane integrity. To examine the specific role of intramitochondrial Zn^2+^ accumulation, the experiment was conducted using blockers (Ruthenium Red) of the mitochondrial Ca^2+^ uniporter channel, through which both Zn^2+^ and Ca^2+^ are able to enter the mitochondrial matrix. At physiological concentrations of extracellular Ca^2+^, the presence of a blocker of the uniporter system accelerated Ca^2+^ deregulation, most likely interrupting mitochondrial Ca^2+^ flux and thus accelerating the lethal accumulation of cytosolic Ca^2+^. In parallel studies, using the ROS indicator, hydroethidine, lowering Ca^2+^ surprisingly accelerated ischemic-induced ROS generation, and under these low Ca^2+^ conditions, either Zn^2+^ chelation or uniporter blockade slowed ROS generation. These studies suggest that, during acute ischemia, Zn^2+^ entry into the mitochondria via the uniporter system induces mitochondrial dysfunction (including ROS generation) occurring upstream and contributes to terminal Ca^2+^ deregulation [60].

Further evidence of the link between Ca^2+^/Zn^2+^ and H_2_O_2_ derives from experiments in rats chronically treated with aldosterone and salt. Under these conditions, a transition of extra- and intracellular Ca^2+^ and intrinsically coupled Zn^2+^ occurs, which the authors call dyshomeostasis, altering the redox state of cardiomyocyte mitochondria, with Ca^2+^ serving as a pro-oxidant and Zn^2+^ as an antioxidant. In fact, compared to controls, results indicate that treatment leads to (a) an increase in both intracellular and mitochondrial Ca^2+^ and Zn^2+^ concentrations; (b) increased production of H_2_O_2_, malondialdehyde, and oxidized glutathione in the mitochondria that coincided with increased Cu/Zn superoxide dismutase and glutathione peroxidase activities; and (c) increased expression of metallothionein-1.

**Figure 4:**
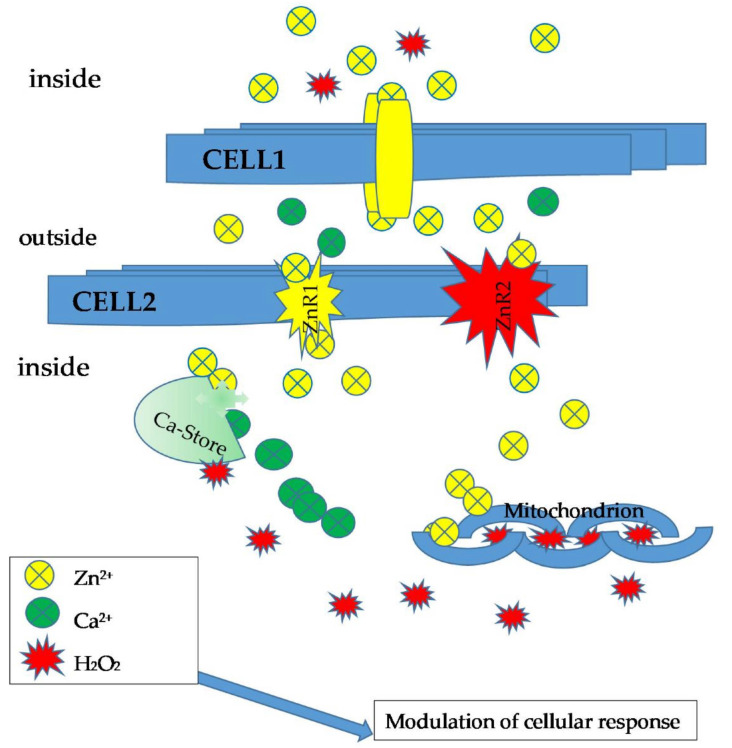
Diagram of extracellular Zn^2+^ signaling pathways. Zn^2+^ released into the extracellular space between cells can interact with receptors active on the surface of another cell through paracrine mechanisms. The binding of the cell to extracellular Zn^2+^ can initiate a variety of responses, including modulation of Ca^2+^ and/or ROS transient (mainly H_2_O_2_), from sites of deposition and intracellular synthesis.

These data indicate quite clearly that the three-sensor system (Ca^2+^, Zn^2+^, and H_2_O_2_) is intrinsically coupled [55] (Figure 4).

## 10. Can Skeletal Muscle Be a Model for Testing the Hypothesis?

The regulation of the force-generating capacity of skeletal muscle can be used to test the possibility that a mechanism set up on the crosstalk of several sensors working in synergy can justify many of the aspects connected with the activation of this particular tissue.

It is known that the cytoplasmic availability of Ca^2+^ required for skeletal muscle fiber contraction is directly linked to the presence of stores (terminal cisternae) containing the ion bound to a protein with a high capacity and low affinity (Calsequestrin) for it. As a result of the propagation of depolarization and activation of the excitation–contraction cycle, Ca^2+^-specific channels (RyR) present in particular structures of the sarcoplasmic reticulum (RS) called triads are opened, resulting in Ca^2+^-ion escaping from the reticular lumen into the sarcoplasm. This causes a 100-fold increase in cytoplasmic Ca^2+^ concentration and the subsequent triggering of force-generating filament sliding. The system returns to its initial state when a specific pump (SERCA) brings Ca^2+^ back into the RS [61].

However, in order to perform its function, the RyR channel needs the coordinated and synergistic presence of other regulatory factors since it can assume, according to different environmental specifications, three different functional states: closed, open, and inactivated. The first and last states prevent Ca^2+^ from escaping the cisterns, while only the second conformation allows this. In addition to the presence of regulatory proteins, such as HRC, triadin, S100/Calmodulin, etc. [62], there are fundamental elements of the luminal and/or sarcoplasmic environment, such as the concentrations in the environment surrounding the channel of Ca^2+^, Zn^2+^, and H_2_O_2_, that guide the presence of channels in one rather than the other of the indicated states.

Using alkaloids, such as caffeine, Ca^2+^ release from the reticulum can be achieved by inducing, in skeletal muscle, a state of prolonged contraction similar to the early stages of fatigue and known as contracture through a mechanism known as Ca^2+^-induced- Ca^2+^-release (CICR). Under these conditions, in rat skeletal muscle, if Zn^2+^ is added to the experimental medium at concentrations of 2–100 μM, a marked and dose-dependent inhibition of alkaloid-induced force generation is observed. At higher concentrations, however, caffeine contracture is enhanced. This means that the effect of Zn^2+^ cannot be explained by the depletion of intracellular deposits alone [63]. Data obtained at the same time show that Zn^2+^ has a biphasic modulatory effect on Ca^2+^ regulatory sites on RYR (the channel does not pass into the active state unless a nmol concentration of Ca^2+^ is present in its niche; it becomes inactive if Ca^2+^ is present in concentrations greater than 1 μM). It should also be noted that the affinity of Ca^2+^ on the binding sites changes significantly in the presence of Zn(^2+^), whereas changes in Ca^2+^ had no clear effect on the affinity of Zn^2+^ on binding [64].

A 170 kDa histidine-rich calcium-binding protein (HRC) residing in the luminal face of the junctional RS is a known regulator of Ca^2+^ uptake, storage, and release by terminal cisternae. It has binding sites for both Ca^2+^ with high capacity, but low affinity and, in a different position, Zn^2+^ binding to the protein induces the loss of affinity of HRC for Ca^2+^ [65,66].

In this situation, which is certainly not exhaustive, what role does the oxidation state of the channel created by changes in H_2_O_2_ concentrations play?

It can be assumed that skeletal muscle generates ROS following physiological contraction and skeletal muscle homogenates contain a constitutively active non-phagocytic NAD(P)H oxidase complex that generates superoxide anion, which is readily converted to H_2_O_2_ [67].

The mitochondria of skeletal muscle fibers are arranged with three different distributions depending on the fiber phenotype examined [68]. In particular, in slow fibers, there is a group of mitochondria distributed in two longitudinal groups, one of which is subsarcolemmal, especially near the capillary network, evidently linked to oxidative phosphorylation and O_2_ uptake, while a second category is arranged transversely, forming two rows on either side of the Z-line [69].

On the other hand, in non-oxidative fast fibers, the group of transversely located mitochondria is closely associated with terminal cisterns and EC cycle sites through small bridges called theters [70]. In these fibers, transversal mitochondria are directly involved in the regulation of sarcoplasmic Ca^2+^ concentration control.

In their recent review, Michelucci et al. (2021) reported “a broad overview of the literature supporting a role for impaired Ca^2+^ handling, dysfunctional Ca^2+^-dependent production of reactive oxygen/nitrogen species (ROS/RNS), and structural/functional alterations in CRUs and mitochondria in the loss of muscle mass, reduction in muscle contractility, and increase in muscle damage in sarcopenia and a wide range of muscle disorders including muscular dystrophy, rhabdomyolysis, central core disease, and disuse atrophy”. However, the collected data are not able to add significant differences in the correlation between Ca^2+^ and H_2_O_2_, to which Zn^2+^ can also be added for all that we have just said and the fiber type analyzed.

Experiments in skinned fibers show that exogenously added H_2_O_2_ stimulates caffeine-induced contraction, clearly supporting ROS activation of CICR. In contrast, the addition of H_2_O_2_ does not affect depolarization-generated contractions. Furthermore, whereas in vitro H_2_O_2_ activates RyR-mediated Ca^2+^ release, H_2_O_2_ activates contraction in skinned fiber without producing an increase in [Ca^2+^]_i_ or even after causing a decrease in [Ca^2+^]_i_ [71].

There is extensive information in the literature demonstrating that RyR possess certain cysteine residues that are highly susceptible to oxidative modifications with considerable effects on RyR function. This propensity has led some authors to propose that RyR channels act as intracellular oxygen or redox sensors [72]. RYR channels consist of four protein subunits containing about 100 cysteine residues each; of these, about 10 to 12 are highly sensitive to oxidation/modification by exogenous sulfhydryl (SH) reagents [67]. Oxidizing and nitrosylating reagents have different effects on single RyR channel activity, which depend on the type of modifying reagent, the RyR isoform, and the ligands bound to the channel. However, based on rather complex calculations, it appears that in each RYR, there are at least nine cysteine residues per subunit capable of influencing (positively or negatively) channel gating [73]. This means that if the cysteine residues are weakly oxidized, the channel is closed; following increasing but not overstated oxidation, the channel is activated, and if oxidation continues, the channel is inactivated; perhaps in this way, the phenomenon of fatigue caused by a state of distress and excessive oxidation/reduction in the so-called vicinal thiol groups (VGT) present on SERCA could also be justified [74].

In conclusion, the proposed crosstalk system for Ca^2+^, Zn^2+^, and H_2_O_2_ could find fertile ground in the management of the excitation–contraction-release cycle of skeletal muscle.

## 11. Conclusions

Without a doubt, H_2_O_2_, Ca^2+^, and Zn^2+^ are mutually interconnected. Ions can increase the production of ROS and, in particular, H_2_O_2_. On the other hand, ROS can significantly influence the influx of these ions into the cell and intracellular stores, using mainly the mitochondria as an exchange station.

All this indicates with reasonable certainty and supported by the abundance of data in the literature that the crosstalk between H_2_O_2_/Ca^2+^/Zn^2+^ is capable of responding adequately to the cellular need.

However, it is not possible to understand whether it is conceivable that the three actors, interacting with each other in different ways, are able to “anticipate” cellular needs. However, the matter is less complicated than it may appear since each small change related to the previous activity alters the distribution of the signalers. Thus, it indicates to the system the new situation that has arisen due to what has just happened. For this reason, a second input that is the same as the previous one may give rise to a different response, even if the perturbing cause remains the same.

This perhaps does not anticipate the cellular necessity but certainly adapts it to the functional state that is present at that moment.

It is rather complicated to think that the various cellular compartments are so finely regulated that they can act as receptors for even small environmental variations. It is more logical to think that only some parts of the complex cellular system have the sensitivity to receipt the smallest variations in the distribution of messengers. Furthermore, it is reasonable to suppose that they are also capable of making their effect felt by behaving as a true “command center” of all cellular activity. Moreover, they are able to modify, even with an ex-novo synthesis, the regeneration of the sensors themselves, making the system more responsive to the new functional state. Therefore, the mitochondrion, endowed with a refined and precise regulation, is able to influence the entire cellular activity and also has the capacity to regulate the presence and levels of the “sensors” (H_2_O_2_, Ca^2+^, Zn^2+^). So, it can be considered the heart of the entire system.

We are aware that what we have tried to make explicit is, above all, a working hypothesis, even if it is deduced from a great deal of experimental data. To substantiate this assertion, we further analyzed ion/ROS crosstalk in skeletal muscle centered on the regulation of RYR and thus its ability to generate force in the striated fiber.

In our opinion, this analysis could provide a rationale for a better understanding of the role that ROS, pivot ions, and mitochondria play in determining the best possible response according to the needs and functional state of the cell.
